# Low-Grade Inflammation and Spinal Cord Injury: Exercise as Therapy?

**DOI:** 10.1155/2013/971841

**Published:** 2013-03-05

**Authors:** Eduardo da Silva Alves, Valdir de Aquino Lemos, Francieli Ruiz da Silva, Fabio Santos Lira, Ronaldo Vagner Thomathieli dos Santos, João Paulo Pereira Rosa, Erico Caperuto, Sergio Tufik, Marco Tulio de Mello

**Affiliations:** ^1^Departamento de Psicobiologia, Universidade Federal de São Paulo, UNIFESP, Campus São Paulo, 04020-050 São Paulo, Brazil; ^2^Centro de Estudos em Psicobiologia e Exercício, (CEPE), 04020-050 São Paulo, Brazil; ^3^Laboratório de Fisiologia e Bioquímica do Exercício, Programa de Pós-Graduação em Ciências da Saúde, Universidade do Extremo Sul Catarinense, Criciúma, SC, Brazil; ^4^Departamento de Biociências, Universidade Federal de São Paulo, UNIFESP, Campus da Baixada Santista, Brazil; ^5^Laboratório do Movimento Humano, Universidade São Judas Tadeu, 04020-050 São Paulo, Brazil

## Abstract

An increase in the prevalence of obesity in people with spinal cord injury can contribute to low-grade chronic inflammation and increase the risk of infection in this population. A decrease in sympathetic activity contributes to immunosuppression due to the lower activation of immune cells in the blood. The effects of physical exercise on inflammatory parameters in individuals with spinal cord injury have not been well described. We conducted a review of the literature published from 1974 to 2012. This review explored the relationships between low-grade inflammation, spinal cord injury, and exercise to discuss a novel mechanism that might explain the beneficial effects of exercise involving an increase in catecholamines and cytokines in people with spinal cord injury.

## 1. Background

Low-grade inflammation is an immune system response that occurs when the body detects injurious stimuli. There can be a neuroendocrine response, such as fever, a blood response, and a metabolic response. White blood cells and cytokines work together to fight against the injury. Interleukins  and  tumor necrosis factor-alpha (TNF-*α*) are important cytokines that play a role in a wide variety of inflammatory reactions. Wang et al. [1] demonstrated that spinal cord injury was associated with increased serum concentrations of C-reactive protein (CRP, a marker of inflammation in the acute phase), interleukin 6 (IL-6), and endothelin-1. These changes suggest that spinal cord injury, independent of obesity, is associated with a state of chronic inflammation, and this association may explain, at least in part, the increase in the atherogenic risk in these patients [1].

With increasing age and time since injury, many individuals with spinal cord injury have an inactive lifestyle, which is associated with deconditioning and secondary health conditions (e.g., osteoporosis, upper extremity pain, obesity, diabetes, and cardiovascular disease), resulting in reduced participation and quality of life [2]. An increase in the prevalence of obesity in people with spinal cord injury can further contribute to low-grade chronic inflammation and increase the risk of cardiovascular disease and type 2 diabetes in this population [3]. Obesity is characterized by an excessive increase in the amount of adipose tissue, which acts as an organ secreting a large variety of proteins, including the proinflammatory cytokines interleukin-6 (IL-6) and TNF-*α* [4].

However, other factors in addition to obesity may contribute to the development of low-grade chronic inflammation in this population. For example, spinal cord injury causes a decrease in sympathetic activity, leading to significant changes in the metabolism of carbohydrates and lipids, such as decreased HDL cholesterol and increased insulin resistance [5]. This decrease in sympathetic activity also contributes to immunosuppression due to the lower activation of immune cells in the blood.

Exercise is an important therapy for the rehabilitation of individuals with spinal cord injury [6, 7]. This is the first to simultaneously discuss the mechanisms related to low-grade inflammation and spinal cord injury, and we propose exercise as a therapy. Our hypothesis is that exercise increases the sympathetic system, causing an increase in catecholamines and leading to enhanced lipolysis and the activation of immune cells in the blood. These effects lead to a decrease in body fat, particularly visceral fat, and the decreased production of inflammatory cytokines by lymphocytes in blood, leading to a decrease in the chronic inflammation found during low-grade inflammation. Thus, the objective of this work was to explore the relationships between low-grade inflammation, spinal cord injury, and exercise to discuss a novel mechanism that might explain the beneficial effects of exercise, which may include an increase in catecholamines and cytokines in people with spinal cord injury.

## 2. Methods

In this paper, we conducted a systematic and integrative review of the literature using source articles indexed by the ISI database, PubMed, and Medline by searching for books that addressed specific aspects related to low-grade inflammation, spinal cord injury, and exercise therapy which were published between 1974 and 2012.

We used the following search terms: “*inflammation and spinal cord injury*” (*n* = 1607), “*inflammation and exercise*” (*n* = 3566), “*inflammation and immune system*” (*n* = 78611), “*spinal cord injury and cytokines*” (*n* = 1087), “*spinal cord injury and obesity*” (*n* = 154), and “*spinal cord injury and exercise*” (*n* = 1787), for a total of 86812 articles. From this total, we selected 72 articles that were specific to the present topic. All the descriptors were searched by Boolean “*or, and, not*” to obtain various arrangements to maximize the search and the quality of research. No restrictions were made regarding age, gender, or experimental design.

## 3. Spinal Cord Injury 

Worldwide, the incidence of spinal cord injuries is 22 occurrences per million people [8]. In the United States, it is estimated that spinal cord injury occurs in approximately 20% of spinal fractures and that from 10 to 15% of patients presenting with severe neurological damage have considerable morbidity and a 5% mortality rate [7]. Because of the high incidence and cost involved in the diagnosis, treatment, and rehabilitation of these patients, spinal cord injury is a major socioeconomic problem [9].

The term spinal cord lesion refers to any lesion that occurs within the medullary canal of neural elements which results in sensory, motor, and autonomic deficits. The individual may become paraplegic (injury between the lower thoracic vertebrae and the lumbar spine) or quadriplegic (lesion in the high thoracic or cervical spine) depending on the region of the spinal injury. Paraplegic individuals have paralysis of the lower limbs and difficulty in staying seated. In turn, tetraplegia is characterized by paralysis of all four extremities, upper and lower, along the trunk musculature [10].

A spinal cord injury can be caused by trauma, viruses, tumors, and schistosomiasis. Spinal cord injury is one of the most debilitating injuries because it is characterized by limited or no neurological recovery [8]. Several situations can lead to spinal cord injury, including automobile accidents, falls from heights, and injury by firearms and sports injuries [8].

## 4. Spinal Cord Injury and Low-Grade Inflammation 

Spinal cord injury is a classic inflammatory process because the pathophysiology of spinal cord injury is characterized by disruption of the axons and cell membranes, cell death, leukocyte migration, and degradation of the myelin sheath [11, 12]. Factors such as an increase in the proinflammatory activity of immune cells and toxic metabolites released from disrupted cells may induce additional tissue damage. Previous studies have demonstrated that alterations in the immune system in individuals with spinal cord injury may further contribute to a low-grade inflammatory process [13, 14]. Over time, individuals with spinal cord injury also exhibit chronic low-grade inflammation [1]. Wang et al. [1] showed that injury itself was associated with increased serum concentrations of C-reactive protein (CRP, a marker of inflammation in the acute phase), IL-6 and endothelin-1. These changes suggest that spinal cord injury per se is associated with a state of chronic inflammation, which may explain, at least in part, the increase in the atherogenic risk in these patients.

In addition to chronic low-grade inflammation, people with spinal cord injury have lower fitness levels, decreased total body lean mass, decreased energy expenditure, fat accumulation in the lower limbs, and abdominal characteristics that increase the risk of this population developing obesity [15, 16].

## 5. Obesity and Inflammation 

Obesity is characterized by an excessive increase in the amount of adipose tissue [17], and it is a low-grade inflammatory disease. The adipose tissue acts as an organ secreting a large variety of proteins, including the proinflammatory cytokines interleukin-6 (IL-6) and TNF-*α* and the anti-inflammatory proteins adiponectin and IL-10 [18, 19]. An increase in the prevalence of obesity in spinal cord injury patients can further contribute to low-grade chronic inflammation, increasing the risk of cardiovascular disease in this population [3]. Obesity contributes to metabolic dysfunction, including increases in circulating cytokines (IL-6, IL-1, and TNF) [20], a decrease in protective factors, such as adiponectin, and communication between cells in inflammatory and metabolic diseases [21].

Cytokines are proteins that are produced and released by different cells, including leukocytes, muscle cells, and neurons. These proteins can act in a pleiotropic way or in synergy with other substances and can modulate the production of other cytokines [22]. Cytokines function in the regulation of metabolism by influencing hormone secretion, regulating Th1/Th2 immune responses, and inducing inflammatory responses. In the nervous system, they regulate complex neuronal actions and modulate thermoregulation, food intake, and neurobiological patterns during sleep [23].

Interleukin-1 (IL-1) family members, such as IL-1*α*, IL-1*β*, IL-1ra, and IL-18, are produced by various cells, such as lymphocytes, in response to inflammation produced by infection and microbial endotoxins [24]. In addition, an increased IL-1 plasma concentration may cause fever, sickness, increased heart rate, increased blood flow in many vascular beds, and increased sympathetic tone; changes in carbohydrate, fat, and protein metabolism also occur [22–25].

TNF-*α* is mainly produced by macrophages and neutrophils, but other cells, such as lymphocytes, NK cells, endothelial cells, and neural cells, might also have the capacity to produce it [25]. TNF-*α* is produced in response to a wide variety of stimuli, including infections and stimulation by other cytokines or mitogens [24]. TNF-*α* is a potent pleiotropic cytokine due to its ability to activate multiple signal transduction pathways and induce or suppress the expression of a number of genes. In addition, it has potent endogenous pyrogenic properties and may promote changes in the body's physiological temperature [26]. Moreover, tissues that present marked cachexia show high TNF-*α* activity, as observed under catabolic conditions, such as cancer and systemic inflammatory diseases [25]. 

 Cytokines can penetrate the blood-brain barrier (BBB) and act indirectly on the brain by stimulating the production of chemical second messengers that carry information to targets, such as NF-*κ*B and adenosine [27–29]. The hypothesis that cytokines could influence the functions of the nervous system (NS) is based on observations that treatment with cytokines, such as interferon-*γ* (INF-*γ*), promotes neuroendocrine alterations. Other studies have identified receptors for these cytokines in many areas of the brain [26, 30, 31]. Additional studies have shown that an increase in proinflammatory cytokine concentrations promotes a decrease in the transendothelial electrical resistance and an increase in the permeability of the BBB [32]. Finally, it is possible that cytokines may be produced within the brain itself in response to neuronal activity [22].

More recently, several studies have shown the existence of an afferent neural pathway through which inflammation in the peritoneal cavity might influence the brain [11]. Subdiaphragmatic transection of the vagus leads to fever reduction, poor sleep, nocturnal norepinephrine secretion, and hypothalamic IL-1 production induced by lipopolysaccharides (LPS) in the peritoneal cavity [32], thereby validating this hypothesis. These alterations are not due to a reduction in the circulating levels of cytokines or the attenuation of the inflammatory response induced by lipopolysaccharide (LPS) but rather to a defective translation of cytokines in the brain [33].

## 6. Sympathetic Activity and Inflammation 

Norepinephrine and epinephrine are key hormones that prepare the body for one of its most primeval reactions: the “fight or flight” response. Catecholamines increase the contractility and conduction velocity of cardiomyocytes, leading to increased cardiac output and an increase in blood pressure, which leads to increased vascular tone and resistance. This results in an increased “pre-load” in the right atrium, causing the heart rate to drop due to the Starling mechanism. Catecholamines also facilitate breathing (bronchi become dilated) and mobilize the body's metabolic reserves (lipolysis and glycogenolysis) to provide vital energy [34].

 Spinal cord-injured individuals with a cervical or high thoracic lesion experience diminished sympathetic nerve traffic below the level of injury [35], including reduced leg sympathetic nerve activity recorded by microneurography [36], decreased whole-body noradrenaline spillover [37], and the impaired release of adrenaline from the adrenal medulla [38]. Based on these findings, it can be hypothesized that not only do these individuals experience a smaller increase in overall sympathoadrenergic activity during arm exercise but also the sympathetic activity in subcutaneous adipose tissue will be less affected in areas proximal (e.g., the clavicular region) to the injury than distal areas (e.g., the umbilical region). Lower plasma catecholamine responses to arm exercise have been found in individuals with spinal cord injury compared with healthy subjects [39]. In accordance with a differentiated autonomic response, hand heating elicits perspiration above but not below the segmental level of interrupted sympathetic output [40].

 Catecholamines are the main regulators of lipolysis plasma hormones in humans. Catecholamines regulate lipolysis by stimulating *α*-adrenergic receptors and can therefore decrease or increase lipolysis, depending on their concentration and binding affinity of the receptor. During exercise, the increase in circulating catecholamines stimulates lipolysis by activating the *α*-adrenergic receptor [41, 42].

 The brain and the immune system are some of the body's major adaptive systems [34], and they communicate with each other extensively in an attempt to regulate body homeostasis [43]. Key systems involved in this crosstalk are the hypothalamic-pituitary-adrenal (HPA) axis and the autonomic nervous system, which consists of the adrenergic sympathetic nervous system, the vagus-mediated parasympathetic nervous system, and the enteric nervous system [34–44]. Over many decades, an increasing body of evidence has demonstrated that lymphocytes and phagocytes are capable of synthesizing and releasing not only neuropeptides but also neurotransmitters and hormones. Furthermore, these cells have adrenergic and cholinergic functions. Thus, by coexisting in the nervous and immune system, these mediators become the universal language of the neuroendocrine-immune-modulating network [45], which enables the nervous, endocrine, and immune system to regulate and fine-tune their functional responses positively or negatively, thereby allowing the body to rapidly adapt to various changes in internal and external environments. 

We are now beginning to understand that catecholamines are an integral part and potent modulators of these neuro-endocrine-immune/inflammatory interactive networks. Through direct communication via sympathetic nerve fibers that innervate lymphoid organs [45], catecholamines can modulate mouse lymphocyte proliferation and differentiation [46] and the cytokine production of rodent. Th cells [47] and human peripheral blood mononuclear cells (PBMCs) [48]. These interactions are facilitated by adrenergic receptors expressed on murine lymphocytes [47], rat natural killer (NK) cells [49], rodent macrophages and neutrophils [50, 51], and human PBMCs [52]. Consequently, we must better understand the sources, distribution, and roles of catecholamines and their receptors in immunity and inflammation.

## 7. Exercise as an Anti-Inflammatory Therapy

A sedentary lifestyle is a risk factor for diseases, with several clinical studies illustrating a reduction of mortality and morbidity in physically active individuals compared to sedentary individuals [53, 54]. The effects of regular or chronic exercise on basal levels of inflammatory markers have been used to recommend exercise as an anti-inflammatory therapy. According to Kasapis and Thompson [55], a single session of exercise triggers an increase in proinflammatory cytokine release, which is associated with leukocytosis and an increased plasma concentration of CRP. This proinflammatory response to acute exercise is accompanied by a sudden increase in oxidative stress, followed by adaptive mechanisms against inflammation [56]. 

Moreover, a longitudinal study showed that regular training induces a reduction in C-reactive protein levels, suggesting anti-inflammatory action, under several conditions, including insulin resistance and other cardiovascular/cardiometabolic diseases. Regular exercise is also associated with decreased IL-6 and TNF-*α* and an increase in anti-inflammatory substances, such as IL-4 and IL-10 [57], reinforcing the anti-inflammatory nature of exercise [58, 59].

Cytokines are released from not only mononuclear cells but also muscle cells. Starkie et al. showed that physical exercise directly inhibits endotoxin-induced TNF-**α** production in humans, most likely through IL-6 release from exercising muscle [60]. Typically, IL-6 is the first cytokine present in circulation after exercise, followed by an increase in IL-1ra and IL-10 [61]. IL-6 release depends on the intensity and duration of exercise and is directly related to the concentration of catecholamines in the blood. The role of IL-6 and the hypothesis of exercise-induced anti-inflammatory IL-6 release has been recently reviewed [62, 63]. Therefore, IL-6, a multifactorial cytokine, regulates cellular and humoral responses and plays a pivotal role in inflammation, being associated with several pathological conditions as a marker of low-grade inflammation [62, 63]. However, what is even more interesting concerning IL-6, as Fisman and Tenenbaum [62] commented, are the putative beneficial effects it has as an anti-inflammatory factor, which is particularly evident in insulin sensitivity during exercise.

A marked increase in circulating levels of IL-6 after exercise without muscle damage has been a remarkably consistent finding. The magnitude by which plasma IL-6 increases is related to exercise duration, the intensity of effort, the muscle mass involved in the mechanical work, and endurance capacity [63] IL-6 has been indicated as the strongest candidate for a humoral factor released after exercise, working in a hormone-like fashion, in which it is released by the muscle, now viewed as an endocrine organ, to influence other organs [63]. Due to its capacity to stimulate the hypothalamus-pituitary-adrenal axis to produce adrenaline, cortisol and anti-inflammatory cytokines, such as interleukin-4 (IL-4) and interleukin-10 (IL-10), IL-6 also has anti-inflammatory properties [25].

 IL-10 was described as a product of lymphocytes that might inhibit Th1 cytokine production. The IL-10 family consists of five other less-studied cytokines: interleukin-19 (IL-19), interleukin-20 (IL-20), interleukin-22 (IL-22), interleukin-24 (IL-24), and interleukin-26 (IL-26). Several tissues may function as sources of IL-10, including lymphocytes and adipose and skeletal muscle. Additionally, IL-10 has multiple biological activities and affects many different cell types, including monocytes/macrophages, T cells, B cells, NK cells, neutrophils, endothelial cells, and peripheral blood mononuclear cells (PBMCs). IL-10 also acts in the regulation of inflammation because it is produced by adipose and muscle tissues, which are important to the pro-/anti-inflammatory ratio under conditions such as physical exercise, obesity, and inflammatory diseases [64, 65].

## 8. Can Physical Exercise Improve Inflammation in Spinal Cord Injuries?

The protective effect of exercise against diseases associated with chronic inflammation may, to some extent, be ascribed to anti-inflammatory activity. Several studies have shown that markers of inflammation are reduced following longer-term behavioral changes involving reduced energy intake and increased physical activity [58]. The data presented in that study highlighted the idea that the beneficial effect of exercise seems to be related to its ability to decrease inflammatory cytokine levels and/or increase anti-inflammatory cytokines, which might also be true for pathological conditions and physical limitations, such as spinal cord injury.

 In a recent review, Martin et al. [66] showed consistent evidence that exercise and physical training are effective in improving the physical fitness of people with spinal cord injury. These benefits were dependent on the time, intensity, and volume of exercise [66]. Regarding the effects of physical activity, some studies have shown that exercise can promote anti-inflammatory effects. Petersen and Pedersen [59] showed that, in healthy subjects, exercise induced the release of IL-6, which is primarily synthesized in muscle fibers and can be released into the blood stream to stimulate the circulation of other anti-inflammatory cytokines, such as receptor antagonist IL-1 (IL-1RA) and IL-10, and inhibit the production of the proinflammatory cytokine TNF-*α*. Furthermore, IL-6 stimulates lipolysis and fat oxidation, and, after exercise, its concentration will depend mainly on the duration and intensity of exercise performed [67].

 Considering the factors mentioned above, we believe that acute exercise causes an increase in sympathetic activity, and this response is intensity dependent, causing an increase in plasma catecholamines and other hormones, thereby modulating the neuro-immuno-endocrine axis in individuals with spinal cord injury. Thus, it is suggested that increasing catecholamines through physical exercise leads to enhanced lipolysis and the activation of immune cells in the blood. These effects lead to a decrease in body fat, particularly visceral fat, and a decreased production of inflammatory cytokines by lymphocytes in the blood, leading to a decrease in the low-grade inflammation found in this population [59, 68–70].

## 9. Conclusions 

The relationships between low-grade inflammation, spinal cord injury, and exercise as therapy are discussed in the present review. Individuals with spinal cord lesions have a higher risk for obesity and, consequently, low-grade chronic inflammation due to the accumulation of visceral fat and the subsequent increased production of proinflammatory cytokines by adipose tissue. Additionally, the decrease in sympathetic activity observed in this population leads to an attenuation of lipolysis, dyslipidemia, and increased macrophage infiltration in adipose tissue, which contribute to chronic inflammation. 

In contrast, acute exercise causes an intensity-dependent increase in sympathetic activity, leading to an increase in plasma catecholamines and a subsequent increase in IL-6 in the blood. This IL-6 release into the circulation after exercise is followed by an increase in IL-1ra and IL-10 anti-inflammatory cytokines. This increase in sympathetic response leads to enhanced lipolysis and the activation of immune cells in the blood. These effects lead to a decrease in body fat, particularly visceral fat, and the decreased production of inflammatory cytokines by lymphocytes in the blood. Given the evidence cited in the study, we conclude that exercise could be an interesting nonpharmacological therapy to decrease chronic low-grade inflammation.

In this sense, we believe that exercise could be considered a modulator of the neuro-immuno-endocrine axis in spinal cord injury patients, acting as a potential option to improve the quality of life in these individuals.
